# Association of Geriatric Comanagement and 90-Day Postoperative Mortality Among Patients Aged 75 Years and Older With Cancer

**DOI:** 10.1001/jamanetworkopen.2020.9265

**Published:** 2020-08-19

**Authors:** Armin Shahrokni, Amy L. Tin, Saman Sarraf, Koshy Alexander, Steve Sun, Soo Jung Kim, Sincere McMillan, Heidi Yulico, Farnia Amirnia, Robert J. Downey, Andrew J. Vickers, Beatriz Korc-Grodzicki

**Affiliations:** 1 Geriatrics Service, Department of Medicine, Memorial Sloan Kettering Cancer Center, New York, New York; 2 Department of Biostatistics and Epidemiology, Memorial Sloan Kettering Cancer Center, New York, New York; 3 Thoracic Service, Department of Surgery, Memorial Sloan Kettering Cancer Center, New York, New York

## Abstract

**Importance:**

Collaboration between geriatricians and surgeons in the perioperative treatment of older patients has been associated with improved outcomes in several nononcologic specialties. Similar associations may be possible among older patients with cancer.

**Objective:**

To investigate the associations of geriatric comanagement of care for older patients undergoing cancer-related surgical treatment with 90-day postoperative mortality, rate of adverse surgical events, and postoperative use of inpatient supportive care services.

**Design, Setting, and Participants:**

This retrospective cohort study assessed outcomes of patients who received geriatric comanaged care vs those who did not using multivariable logistic regression analysis, with 90-day mortality as the outcome and geriatric comanagement of care as the main variable, with adjustment for age, sex, American Society of Anesthesiology score, Memorial Sloan Kettering Frailty Index score, preoperative albumin level, operative time, and estimated blood loss. A similar model was used to assess the association of geriatric comanagement with adverse surgical events, defined as any major complication, readmission, or emergency department visit within 30 days. Patients aged 75 years and older who underwent an elective surgical procedure with a hospital stay of at least 1 day at a single tertiary-care cancer center between February 2015 and February 2018 were included. Data were analyzed from January to July 2019.

**Exposures:**

Postoperative care comanaged by the geriatrics service and surgical service (geriatric comanagement group) vs by the surgical service only (surgical service group).

**Main Outcomes and Measures:**

90-day mortality, adverse surgical events, and use of supportive care services.

**Results:**

Of 1892 patients included, 1020 (53.9%) received geriatric comanagement of care; these patients, compared with those who received care managed by the surgery service only, were older (mean [SD] age, 81 [4] years vs 80 [4] years; *P* < .001), had longer operative time (mean [SD], 203 [146] minutes vs 138 [112] minutes; *P* < .001), and longer length of stay (median [interquartile range], 5 [3-8] days vs 4 [2-7] days; *P* < .001). There were no differences in the proportions of men (488 [47.8%] men vs 450 [51.6%] men; *P* = .11). Adverse surgical events were not significantly different between groups (odds ratio, 0.93 [95% CI, 0.73-1.18]; *P* = .54). However, the adjusted probability of death within 90 days after surgical treatment was 4.3% for the geriatric comanagement group vs 8.9% for the surgical service group (difference, 4.6% [95% CI, 2.3%-6.9%]; *P* < .001). Additionally, compared with patients who received postoperative care management from the surgery service only, a higher proportion of patients in the geriatric comanagement group received inpatient supportive care services, including physical therapy (555 patients [63.6%] vs 820 patients [80.4%]; *P* < .001), occupational therapy (220 patients [25.2%] vs 385 patients [37.7%]; *P* < .001), speech and swallow rehabilitation (42 patients [4.8%] vs 86 patients [8.4%]; *P* = .002), and nutrition services (637 patients [73.1%] vs 803 patients [78.7%]; *P* = .004).

**Conclusions and Relevance:**

This cohort study found that geriatric comanagement was associated with significantly lower 90-day postoperative mortality among older patients with cancer. These findings suggest that such patients may benefit from geriatric comanagement, which could improve their ability to survive adverse postoperative events.

## Key Points

**Question:**

Is collaboration between geriatricians and surgeons in the perioperative care of older patients with cancer associated with postoperative outcomes?

**Findings:**

In this cohort study including 1892 patients aged 75 years and older, the adjusted probability of death within 90 days after surgery was 4.3% for patients who received geriatric comanagement of care, compared with 8.9% for patients who received care management from the surgical service only.

**Meaning:**

These findings suggest that when feasible, older patients undergoing surgical treatment for cancer should receive geriatric care comanagement as part of their perioperative care.

## Teaser

This cohort study examines outcomes associated with geriatric comanaged perioperative care vs care managed by the surgery team alone among patients aged 75 years and older with cancer.

## Introduction

Older patients with cancer are a heterogenous group, with chronological age being only 1 of several risk factors associated with poor surgical outcomes.^1^ Furthermore, there is a high prevalence of prefrailty and frailty in the population of patients with cancer, with older adults of the same age having different levels of fitness, leading to different outcomes. For example, older patients who are fit may do as well as younger patients, whereas older patients who are frail have an increased risk of adverse postoperative outcomes. In a 2014 study of 180 patients who underwent surgical treatment for gastric cancer,^2^ postoperative mortality was 23% among patients who were frail, compared with 5% among patients who were fit. A 2013 study^3^ reported that, compared with patients in their 80s who were fit, those who were frail were 8.4-fold more likely to die within 1 year after elective colectomy for colon cancer. A 2016 systematic review of 4 studies of the associations of frailty with outcomes after colorectal cancer surgical treatment^4^ confirmed that patients who were frail had less favorable outcomes than those who were fit. Another systematic review that included 23 studies of patients with and without cancer^5^ confirmed that frailty had the strongest correlation with increased risk of mortality at 30 and 90 days, postoperative complications, and hospital length of stay (LOS).

Various strategies have been proposed for improving postoperative outcomes among patients who are older and frail, including enhanced recovery after surgical treatment pathways,^6^ prehabilitation,^7^ and geriatric comanagement of care. Geriatric comanagement of care is characterized by collaboration between geriatrics and nongeriatrics teams, with a focus on the prevention and management of geriatric syndromes and complications.^8^ A key component of geriatric comanagement is the geriatric assessment, which can be used to identify frailty and risk factors among older patients that are not always apparent during general preoperative assessment. The benefits of geriatric comanagement on LOS, complication rates, and mortality have been demonstrated in the care of older patients with hip fractures in both observational and randomized clinical trials.^9,10,11^

Data on the benefits of geriatric comanagement of care for patients with cancer are limited. A 2019 study^12^ found that cytoreductive surgical treatment among older women with advanced ovarian cancer who were frail could be performed safely if perioperative comanagement was used. At present, only a minority of surgeons collaborate in any way with geriatricians in the delivery of perioperative care for older patients with cancer.^13^

In this cohort study, we investigate the association of 90-day postoperative mortality and geriatric comanagement of care for older patients undergoing cancer-related surgical treatment. We also assess the rate of adverse surgical events and determine whether geriatric comanagement is associated with postoperative use of inpatient supportive care services.

## Methods

### Patient Population

This cohort study was approved by the Memorial Sloan Kettering Cancer Center institutional review board with a waiver of informed consent because geriatric comanagement is part of established and routine care at Memorial Sloan Kettering Cancer Center. The data were also deidentified for analysis. Our cohort included retrospectively identified patients aged 75 years and older who underwent cancer-related surgical treatment at Memorial Sloan Kettering Cancer Center between February 2015 and February 2018. The cohort included patients with various tumor types who underwent elective surgical treatment within 60 days of their first visit with the surgeon and required a hospital stay of at least 1 day. In total, 1970 patients met these criteria; owing to a lack of sufficient follow-up, mortality status could not be confirmed for 78 patients. The final cohort comprised 1892 patients (Figure). The cohort was divided into 2 groups: patients whose care was comanaged by the geriatrics service and surgical service (geriatric comanagement group) and patients whose care was managed by the surgical service without involvement of the geriatrics service (surgical service group).

**Figure.**
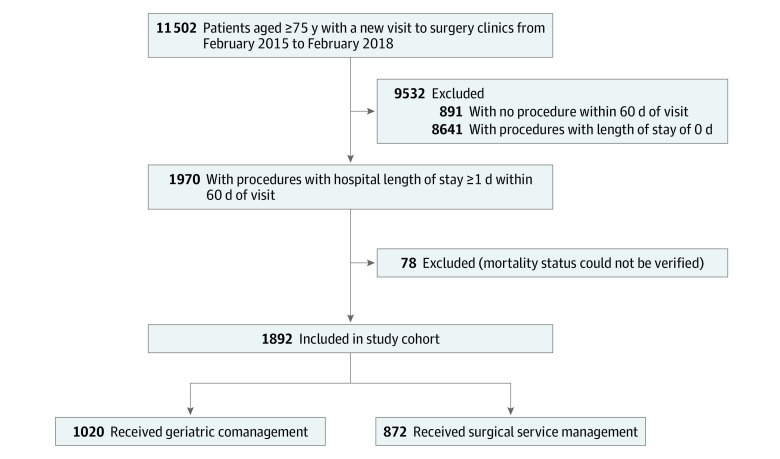
Participant Flow Through the Study

### Geriatric Comanagement

Geriatric comanagement at Memorial Sloan Kettering is performed by the geriatrics service and includes 2 phases: preoperative and postoperative care. At our institution, owing to high patient volumes, only patients aged 75 years and older are eligible for referral to the geriatrics service for preoperative evaluation. In this population, referral to the geriatrics service is based on the surgery team’s preference and clinical judgment. A formal frailty screening tool is not used for referral to the geriatrics service.

### Preoperative Phase

Patients referred to the geriatrics service for preoperative evaluation are evaluated using an electronic form of geriatric assessment called the *electronic Rapid Fitness Assessment*. Details about this assessment have been published elsewhere^14^ and are available in the eAppendix and eTable 1 in the Supplement. Following completion of the assessment and discussion with the surgery and anesthesiology teams, the geriatrics team recommends interventions aimed at optimizing the patient’s status. These recommendations include but are not limited to further consultation with other services, such as cardiology and endocrinology. The geriatrics team also provides patient and caregiver education on a variety of subjects, including the importance of exercise before surgical treatment, the use of incentive spirometry, the postoperative care process, adequate pain management, and delirium risk-reduction interventions. To empower patients and caregivers to research rehabilitation centers even before surgical treatment, the possible need for home care services or subacute rehabilitation at hospital discharge after surgical treatment is also discussed. As necessary, age-based life expectancy is discussed to align expectations of recovery with it.^15^

### Postoperative Phase

All patients seen by the geriatrics service before their operation are followed up after their operation while inpatients, with the geriatrics service in a consultative role. All efforts are made by the geriatrics service to see patients on postoperative day 1 and for the first 3 days after surgical treatment; further follow-up is based on the clinical judgement of the inpatient geriatrics service. The geriatrics team assists the surgical team with the management of comorbid conditions and the reintroduction of medications (eg, antihypertensive medications, in the setting of fluid shifts and volume status changes associated with surgical treatment, and diabetic medications, in the setting of changes in food and fluid intake from baseline). Geriatricians implement interventions aimed at reducing the risk of delirium, such as reviewing medication lists and reducing them as appropriate, discontinuing intravenous lines and Foley catheters in a timely manner, assisting with early mobility, and encouraging functional activity, such as getting patients out of bed or requesting supportive services (eg, physical therapy). The geriatrics team also focuses on recovery of respiratory function by encouraging and educating patients to use incentive spirometry. Attention is also paid to prevention of deep venous thrombosis, which is important for patients with malignant neoplasms and temporary mobility restrictions. Geriatricians ensure that patients’ perioperative pain is well managed, in collaboration with the pain management team, and that patients follow appropriate bowel regimens while receiving narcotic medications. Finally, they assist surgeons with proper hospital disposition. If changes have been made to patients’ usual home medications, this information is reinforced with the patients and their caregivers, and a plan for follow-up with community clinicians is recommended. Outpatient care after hospital discharge is provided only by the surgical teams; this usually consists of a clinic visit 1 to 2 weeks after hospital discharge to assess the patient’s postoperative recovery.

### Surgical Service Management

Patients whose care is managed by the surgical service, without geriatric comanagement, may undergo preoperative evaluation by a nongeriatrician (eg, a cardiologist or internist). A geriatric consultation may be requested during the postoperative inpatient period.

### Outcomes

The primary outcome of interest was 90-day postoperative mortality, which was retrieved from the Social Security Death Index or by electronic medical record review. We hypothesized that patients in the geriatric comanagement group would have lower 90-day mortality than patients in the surgical service group. Secondary outcomes of interest were factors that could contribute to 90-day mortality: rate of adverse surgical events (defined as any major surgical complication [grade ≥3 Clavien-Dindo classification^16^]) and rate of emergency department visits or hospital readmissions within 30 days of surgical treatment. Additional outcomes included inpatient postoperative supportive care use and location to which patients were discharged postoperatively. Use of inpatient postoperative supportive care services was ascertained by reviewing patient electronic medical records to assess whether the patient was evaluated at least once after surgical treatment and before discharge by any of the following services: physical therapy, occupational therapy, speech and swallow, psychiatry or psychology, nutrition, social work, or case management. Postoperative discharge locations included home, home with home supportive services, inpatient hospice or home with hospice, rehabilitation facility, or transfer to another hospital.

### Statistical Analysis

We investigated the association of 90-day mortality with geriatric comanagement by creating a multivariable logistic regression model with 90-day mortality as the outcome and geriatric comanagement as the main variable, adjusted for age at surgical treatment, sex, American Society of Anesthesiology score, preoperative albumin level, operative time, estimated blood loss, and Memorial Sloan Kettering Frailty Index (MSK-FI) score. The development of the MSK-FI has been described elsewhere.^17^ In brief, the MSK-FI was developed at Memorial Sloan Kettering on the basis of *International Classification of Diseases, Ninth Revision *(*ICD-9*)^29^ or* International Statistical Classification of Diseases, Tenth Revision *(ICD-10)^30^ codes and includes 10 comorbid conditions and 1 item related to impairment of the 5 activities of daily living.^18^ Scores for the MSK-FI range from 0 to 11, with higher scores indicating more frailty. We have previously shown that the MSK-FI is associated with the previously validated geriatric assessment and with postoperative outcomes of older patients with cancer.^17^ Preoperative albumin level was measured in specimens drawn within 30 days before surgical treatment. We report adjusted 90-day mortality with corresponding 95% CIs around the difference in rates for the 2 groups.

Next, we assessed the association between geriatric comanagement and the composite outcome of adverse surgical events by creating a second multivariable logistic regression model, with adverse surgical events as the outcome, geriatric comanagement as the main variable, and the same covariates as in the first model. Finally, we created a third multivariable logistic regression model with 90-day mortality as the outcome that included adjustment for adverse surgical events in addition to the covariates in the primary model. We present rates of use of inpatient postoperative supportive care and postoperative discharge location; group comparisons were made using Fisher exact test.

A total of 834 patients (44.1%) were missing at least 1 of the covariates included in our models. The most frequent missing covariate was estimated blood loss (unknown in 490 patients [25.9%]), followed by preoperative albumin level (315 patients [16.6%]), MSK-FI score (205 patients [10.8%]), and American Society of Anesthesiology score (86 patients [4.6%]). Multiple imputation by chained equations was used to impute missing data. All analyses were performed using the measured and imputed values combined across 20 imputations using Rubin’s method. All tests were 2-sided, and significance was set at *P* < .05. All statistical analyses were conducted using Stata statistical software version 15.0 (StataCorp). Data were analyzed from January to July 2019.

## Results

Of 1892 patients included in the analysis, patients in the geriatric comanagement group, compared with those in the surgical service group, were older (mean [SD] age, 81 [4] years vs 80 [4] years; *P* < .001) and had longer operative time (mean [SD], 203 [146] minutes vs 138 [112] minutes; *P* < .001) and longer LOS (median [interquartile range], 5 [3-8] days vs 4 [2-7] days; *P* < .001). There were no differences in the proportions of men (488 [47.8%] men vs 450 [51.6%] men; *P* = .11) or in frailty measured by MSK-FI. Patient characteristics by care management group are shown in Table 1; the proportions of patients referred for geriatric comanagement per procedure type are listed in eTable 2 in the Supplement. Of our cohort, 1020 patients (53.9%) received geriatric comanagement.

**Table 1.** Demographic and Perioperative Characteristics of 1892 Patients

Characteristic	No. (%)		*P* value
	Surgical service management (n = 872)	Geriatric comanagement (n = 1020)	
Age, mean (SD) y	80 (4)	81 (4)	<.001
Men	450 (51.6)	488 (47.8)	.11
Length of stay, median (IQR), d	4 (2-7)	5 (3-8)	<.001
Operative time, mean (SD), min	138 (112)	203 (146)	<.001
ASA score >3^a^	818 (93.8)	963 (94.4)	.62^b^
Preoperative albumin level, mean (SD) g/dL^a^	3.9 (0.5)	4.0 (0.4)	<.001^b^
Estimated blood loss, mean (SD), mL ^a^	138 (309)	200 (298)	<.001^b^
MSK-FI score^a^			
0	104 (11.9)	111 (10.9)	.32^b^
1	227 (26.0)	294 (28.8)	
2	213 (24.4)	278 (27.3)	
3	156 (17.9)	168 (16.5)	
4	98 (11.2)	95 (9.3)	
≥5	74 (8.5)	74 (7.3)	
Procedure type^c^			
Colorectal	137 (15.7)	448 (43.9)	<.001
Gastric and mixed tumor	92 (10.6)	82 (8.0)	.07
Gynecology	45 (5.2)	264 (25.9)	<.001
Head and neck	147 (16.9)	314 (30.8)	<.001
Urology	78 (8.9)	180 (17.6)	<.001
Plastic	47 (5.4)	118 (11.6)	<.001
Hepatobiliary-pancreatic	55 (6.3)	183 (17.9)	<.001
Thoracic	309 (35.4)	150 (14.7)	<.001
Other procedures	109 (12.5)	159 (15.6)	.06

Abbreviations: ASA, American Society of Anesthesiologists; IQR, interquartile range; MSK-FI, Memorial Sloan Kettering Frailty Index.

SI conversion factor: To convert albumin to grams per liter, multiply by 10.

^a^ Data for characteristics with incomplete data are shown combined across 20 imputations using Rubin’s method.

^b^ *P* values assessing the association between geriatric comanagement and characteristics with incomplete data were assessed using univariable logistic regression and combined across 20 imputations using Rubin method.

^c^ Percentages do not sum up to 100, as patients underwent multiple procedures under different types.

### Mortality

Of 1892 patients, 128 died within 90 days of surgical treatment, including 36 death in the geriatric comanagement group and 92 deaths in the surgical service group (unadjusted 90-day mortality, 3.5% vs 10.6%). On multivariable logistic regression analysis, patients in the geriatric comanagement group were less likely to die within 90 days after surgical treatment (odds ratio [OR], 0.43 [95% CI, 0.28-0.67]; *P* < .001). The adjusted probability of death within 90 days of surgical treatment was 8.9% in the surgical service group vs 4.3% in the geriatric comanagement group (difference, 4.6% [95% CI, 2.3%-6.9%]; *P* < .001), which leads to a number needed to treat of 22. After adjustment for adverse surgical outcomes in addition to the covariates in our primary model, 90-day mortality remained lower in the geriatric comanagement group (OR, 0.44 [95% CI, 0.28-0.68]; *P* < .001).

### Adjusted Mortality

In sensitivity analyses, all risk of 90-day mortality in the geriatric comanagement group was still statistically significantly lower compared with the surgery service group (eTable 3 in the Supplement). The incorporation of procedure type increased the risk the most (OR, 0.57 [95% CI, 0.36-0.90]) (eTable 4 in the Supplement). In a final, fully adjusted model that included multiple variables adjusted for in the sensitivity analyses but not in the primary model, geriatric comanagement was significantly associated with reduced 90-day mortality (OR, 0.58 [95% CI, 0.37-0.92]) (eTable 4 in the Supplement).

### Adverse Surgical Outcomes

Adverse surgical outcomes were experienced by 401 patients. Unadjusted rates of adverse surgical outcomes are listed in eTable 5 in the Supplement. Adverse surgical outcomes within 30 days of surgical treatment did not differ on the basis of management group (OR, 0.93 [95% CI, 0.73-1.18]; *P* = .54). The probability of adverse surgical outcomes, adjusted for the covariates in our primary logistic regression model, was 21.8% in the surgical service group vs 20.6% in the geriatric comanagement group (difference, 1.2% [95% CI, -2.7% to 5.1%]; *P* = .54).

### Support Services and Discharge Location

Rates of physical therapy, occupational therapy, speech and swallow rehabilitation, and nutrition services were higher in the geriatric comanagement group (Table 2). A higher proportion of patients in the geriatric comanagement group were discharged home with home supportive services such as a visiting nurse (182 patients [18.0%] vs 115 patients [13.6%]; *P* < .001) (Table 3).

**Table 2.** Use of Inpatient Supportive Care Services for 1892 Patients

Service	No. (%)		*P* value^a^
	Surgical service management (n = 872)	Geriatric comanagement (n = 1020)	
Physical therapy	555 (63.6)	820 (80.4)	<.001
Occupational therapy	220 (25.2)	385 (37.7)	<.001
Speech and swallow rehabilitation	42 (4.8)	86 (8.4)	.002
Psychiatry	41 (4.7)	55 (5.4)	.53
Nutrition	637 (73.1)	803 (78.7)	.004
Social work	166 (19.0)	208 (20.4)	.49
Case management	623 (71.4)	768 (75.3)	.06

^a^ Group comparisons were made using Fisher exact test.

**Table 3.** Discharge Locations Among 1855 Patients Who Survived to Hospital Discharge

Discharge location	No. (%)		*P* value^a^
	Surgical service management (n = 846)	Geriatric comanagement (n = 1009)	
Home	676 (79.9)	731 (72.4)	<.001
Home with home supportive services	115 (13.6)	182 (18.0)	
Hospice or home with hospice	8 (0.9)	2 (0.2)	
Rehabilitation facility	44 (5.2)	89 (8.8)	
Transfer to another hospital	3 (0.4)	5 (0.5)	

^a^ Group comparison made using Fisher exact test.

## Discussion

This cohort study found that older patients whose care was comanaged by the geriatrics and surgical services had significantly lower 90-day postoperative mortality than patients whose care was managed by the surgical service only. This finding remained significant after adjustment for confounding variables, such as age, sex, American Society of Anesthesiology score, operative time, preoperative albumin level, estimated blood loss, frailty, procedure type, cardiac disease, and preoperative blood test results (eg, hemoglobin, sodium, and calcium levels). The lower mortality among patients whose care was comanaged is consistent with observations in the nononcologic surgery literature. A systematic review of 18 studies (1 randomized clinical trial and 6 retrospective, 7 prospective, and 4 prospective cohort retrospective control studies)^8^ found that geriatric comanagement was associated with lower in-hospital mortality (relative risk, 0.60 [95% CI, 0.43-0.84]) and long-term mortality (relative risk, 0.83 [95% CI, 0.74-0.94]). Another literature review (including 21 studies, 8 of which were randomized)^19^ found that integrated orthopedic and geriatric care was associated with better short- and long-term mortality, compared with orthopedic care only. To our knowledge, our study is the first to assess the association of 90-day postoperative mortality with geriatric comanagement.

Geriatric comanagement can reduce postoperative mortality by various mechanisms. In our study, more patients who received comanaged care received inpatient supportive care services. The difference in use of supportive care services continued after hospital discharge. Such services may play a role in attenuating the adverse outcomes associated with surgical stress on these patients. Future studies should assess how often such services are used and their effects on outcomes in the perioperative period. Other potential mechanisms warrant further study. A recent nonsurgical study in older adults with cancer^20^ found that geriatric assessment was associated with an increased number of conversations about aging-related concerns; therefore, the content and quality of perioperative communications between health care practitioners and older patients with cancer and their caregivers should be investigated. The geriatrics service at our institution emphasizes the need for daily incentive spirometry to patients and their caregivers. Surgical team members may recommend this less strongly. A 2019 randomized clinical trial^21^ of 160 patients who underwent coronary artery bypass graft showed that a simple reminder to patients to use incentive spirometry reduced in-hospital mortality from 4% to 1%. Another possible mechanism underlying the lower postoperative mortality associated with geriatric comanagement is the degree of treatment optimization of comorbid conditions and the management of polypharmacy in the perioperative period. At our institution, the geriatrics service discusses the risks and benefits of sedative medications (eg, benzodiazepines) with patients and their caregivers. Unless patients are using these medications chronically, we recommend these medications not be used in the perioperative period.^22^ Such interventions may be associated with a decrease in postoperative delirium, which is shown to be associated with postoperative mortality.^23,24^

Finally, the lower rate of 30-day emergency department visits or hospital readmissions may be explained by a reduction in surgical complications or improvement in detection and management of surgical complications associated with geriatric comanagement. A 2014 study^25^ reported that occurrence of major complications was associated with a higher likelihood of 30-day hospital readmissions and emergency department visits. Surgical complications, emergency department visits, and hospital readmissions have all been associated with mortality.^26,27,28^ In our study, the combination of a higher rate of major complications and a lower rate of emergency department visits and readmissions in the geriatric comanagement group leads to various hypotheses. One hypothesis is that complications were identified earlier during hospitalization in the geriatric comanagement group. This may explain the higher rate of diagnosis and management of complications in this group, which then led to lower rates of emergency department visits and hospital readmissions. Nonetheless, future prospective studies with more homogenous cohorts of older patients with cancer are needed to assess the validity of such hypotheses.

### Limitations and Strengths

Our study has several limitations. First, this was not a randomized clinical trial. Patients were referred to the geriatrics service or to the community primary care practitioner or subspecialist for preoperative evaluation mainly on the basis of the surgeon’s or patient’s preference. Second, our study included a heterogenous mix of malignant neoplasms and surgical procedures. We lack information on preoperative systemic or radiation treatments patients may have received. We also do not have data on cancer stage; however, we anticipate that the effect of cancer stage on short-term mortality following surgical treatment would be small. The exact details of the perioperative care process, such as treatment optimization of comorbid conditions, additional consultations and tests requested by the geriatrics service vs the surgery team, and specific interventions in the postoperative period, are beyond the scope of this study and will be assessed in future studies comprising better-defined groups of patients. We assessed only readmissions and emergency department visits to the index hospital. Readmissions and emergency department visits to other health care institutions may not have been documented. Additionally, we did not have data on other important outcomes, such as functional recovery after surgical treatment or ability to receive additional cancer treatment within 90 days after surgical treatment.

Our study also has several strengths. To our knowledge, this is the largest study to assess the role of geriatric comanagement in patients aged 75 years and older who underwent surgical treatment for cancer. The study spanned 3 years (2015-2018), reducing the possibility that advances in surgical techniques or implementation of surgical quality-improvement initiatives influenced outcomes. The short duration of the study also limits the possible associations of other surgical quality-improvement initiatives with outcomes.

## Conclusions

This cohort study found that geriatric comanagement was associated with a significant reduction in 90-day postoperative mortality among older patients with cancer. Geriatric comanagement may improve patients’ ability to survive adverse postoperative events. This finding should be assessed in the future by conducting randomized clinical trials.

## Notes

Supplement. **eAppendix.** Electronic Rapid Fitness Assessment and MSK Geriatric Assessment **eTable 1.** The Electronic Rapid Fitness Assessment Instruments **eTable 2.** Proportion of Patients Referred for Geriatric Comanagement Based on Procedure Type **eTable 3.** Association Between Geriatric Comanagement and Mortality on Sensitivity Analyses **eTable 4.** Association Between Geriatric Comanagement and 90-day Mortality on Sensitivity Analyses, With Adjustment for Additional Covariates **eTable 5.** Adverse Surgical Events Within 30 Days of Surgical Treatment
