# Supplementary material for: Association of Geriatric Comanagement and 90-Day Postoperative Mortality Among Patients Aged 75 Years and Older With Cancer
Source: JAMA Netw Open. 2020 Aug 19;3(8):e209265. doi: 10.1001/jamanetworkopen.2020.9265 (PMC7439108; doi:10.1001/jamanetworkopen.2020.9265)
Supplement: Supplement. — eAppendix. Electronic Rapid Fitness Assessment and MSK Geriatric Assessment eTable 1. The Electronic Rapid Fitness Assessment Instruments eTable 2. Proportion of Patients Referred for Geriatric Comanagement Based on Procedure Type eTable 3. Association Between Geriatric Comanagement and Mortality on Sensitivity Analyses eTable 4. Association Between Geriatric Comanagement and 90-day Mortality on Sensitivity Analyses, With Adjustment for Additional Covariates eTable 5. Adverse Surgical Events Within 30 Days of Surgical Treatment [file jamanetwopen-3-e209265-s001.pdf]

Supplementary Online Content

Shahrokni A, Tin AL, Sarraf S, et al. Association of Geriatric Comanagement and 90-Day Postoperative Mortality Among Patients Aged 75 Years and Older With Cancer. *JAMA Netw Open*. 2020;3(8):e209265. doi:10.1001/jamanetworkopen.2020.9265

eAppendix. Electronic Rapid Fitness Assessment and MSK Geriatric Assessment
eTable 1. The Electronic Rapid Fitness Assessment Instruments
eTable 2. Proportion of Patients Referred for Geriatric Comanagement Based on Procedure Type
eTable 3. Association Between Geriatric Comanagement and Mortality on Sensitivity Analyses
eTable 4. Association Between Geriatric Comanagement and 90-day Mortality on Sensitivity Analyses, With Adjustment for Additional Covariates
eTable 5. Adverse Surgical Events Within 30 Days of Surgical Treatment

This supplementary material has been provided by the authors to give readers additional information about their work.

© 2020 Shahrokni A et al. *JAMA Network Open.*

eAppendix. Electronic Rapid Fitness Assessment and MSK Geriatric Assessment

Since 2015, the eRFA, a web-based geriatric assessment, has become the standard of care for all older adults evaluated in our Geriatrics Service clinics at MSK. The instruments of the eRFA are listed in Supplementary Table 1. Patients and/or their caregivers are responsible for completing all but two instruments of the eRFA. Geriatrics registered nurses perform a cognitive assessment using the Mini-Cog[1] and assess gait speed using the Timed Up and Go test.[2] The final eRFA report is available for review before the geriatrician sees the patient. The report is also included in the final preoperative clearance note.

Sensitivity Analyses

The effect size in our primary analysis was larger than anticipated, with patients in the geriatric comanagement group having a >50% lower odds of dying within 90 days, compared with the surgical management group. This warranted further investigation to determine whether there were measured confounders not included in our primary analysis. We first evaluated whether variables in our data set were associated with 90-day mortality and geriatric comanagement. Results from these exploratory analyses prompted the following *post hoc* sensitivity analyses, with additional adjustment for (i) time from initial visit with the surgeon to surgery (to account for semi urgent surgeries), (ii) other preoperative blood measures (hemoglobin, sodium, calcium), (iii) cardiac disease and arthritis or degenerative joint disease, and (iv) surgical procedure type. For this last analysis, there were 10 surgical categories in our data set (Table 1). To prevent our model from being overfit, we used a two-step model. We first created a multivariable model with 90-day mortality as the outcome and procedure type as the predictor and derived the linear predictor from this model. We then used the model as defined in the primary analysis and additionally adjusted for the linear predictor. We additionally included a sensitivity analysis with 30- and 60-day mortality as the outcomes. Due to the limited number of deaths within 30 days, the covariates adjusted for the outcome of 30-day mortality were limited to age at surgery, preoperative albumin level, operative time, and MSK-FI score. The covariates for the outcome of 60-day mortality were the same covariates as in our primary analysis (eTables 3 and 4).

© 2020 Shahrokni A et al. *JAMA Network Open.*

References

1. Borson S, Scanlan J, Brush M, Vitaliano P, Dokmak A. The mini-cog: a cognitive 'vital signs' measure for dementia screening in multi-lingual elderly. *Int J Geriatr Psychiatry.* 2000;15(11):1021-1027.
2. Podsiadlo D, Richardson SJ. The timed “Up & Go”: a test of basic functional mobility for frail elderly persons. *J Am Geriatr Soc*. 1991;39(2):142-148..
3. Hartigan I. A comparative review of the Katz ADL and the Barthel Index in assessing the activities of daily living of older people. *Int J Older People Nurs.* 2007;2(3):204-212.
4. Katz S, Downs TD, Cash HR, Grotz RC. Progress in development of the index of ADL. *Gerontologist.* 1970;10(1):20-30.
5. Katz S, Ford AB, Moskowitz RW, Jackson BA, Jaffe MW. Studies of illness in the aged. The index of ADL: a standardized measure of biological and psychosocial function. *JAMA.* 1963;185:914-919.
6. Hoppe S, Rainfray M, Fonck M, et al. Functional decline in older patients with cancer receiving first-line chemotherapy. *J Clin Oncol.* 2013;31(31):3877-3882.
7. Lawton MP, Brody EM. Assessment of older people: self-maintaining and instrumental activities of daily living. *Gerontologist.* 1969;9(3):179-186.
8. Mor V, Laliberte L, Morris JN, Wiemann M. The Karnofsky Performance Status Scale. An examination of its reliability and validity in a research setting. *Cancer.* 1984;53(9):2002-2007.
9. Guideline for the prevention of falls in older persons. American Geriatrics Society, British Geriatrics Society, and American Academy of Orthopaedic Surgeons Panel on Falls Prevention. *J Am Geriatr Soc.* 2001;49(5):664-672.
10. Bohannon RW. Reference values for the timed up and go test: a descriptive meta-analysis. *J Geriatr Phys Ther.* 2006;29(2):64-68.
11. Yeung TS, Wessel J, Stratford PW, MacDermid JC. The timed up and go test for use on an inpatient orthopaedic rehabilitation ward. *J Orthop Sports Phys Ther.* 2008;38(7):410-417.
12. Gnjidic D, Hilmer SN, Blyth FM, et al. Polypharmacy cutoff and outcomes: five or more medicines were used to identify community-dwelling older men at risk of different adverse outcomes. *J Clin Epidemiol.* 2012;65(9):989-995.
13. Gjesfjeld CD, Greeno CG, Kim KH. A confirmatory factor analysis of an abbreviated social support instrument: the MOS-SSS. *Res Soc Work Pract.* 2008;18(3):231-237.
14. Sullivan M. Measuring Functioning and Well-Being - the Medical Outcomes Study Approach - Stewart,Al, Ware,Je. *Psycho-Oncology.* 1995;4(2):163-165.
15. Pomeroy IR, Clark CR, Philp I. The effectiveness of very short scales for depression screening in elderly medical patients. *Int J Geriatr Psychiatry.* 2001;16(3):321-326.
16. Hegel MT, Collins ED, Kearing S, Gillock KL, Moore CP, Ahles TA. Sensitivity and specificity of the Distress Thermometer for depression in newly diagnosed breast cancer patients. *Psychooncology.* 2008;17(6):556-560.
17. Roth AJ, Kornblith AB, Batel-Copel L, Peabody E, Scher HI, Holland JC. Rapid screening for psychologic distress in men with prostate carcinoma: a pilot study. *Cancer.* 1998;82(10):1904-1908.
18. Mitchell AJ. Pooled results from 38 analyses of the accuracy of distress thermometer and other ultra-short methods of detecting cancer-related mood disorders. *J Clin Oncol.* 2007;25(29):4670-4681.
19. Ransom S, Jacobsen PB, Booth-Jones M. Validation of the Distress Thermometer with bone marrow transplant patients. *Psychooncology*. 2006;15(7):604-612.
20. Holland JC, Bultz BD, National Comprehensive Cancer Network. The NCCN guideline for distress management: a case for making distress the sixth vital sign. *J Natl Compr Canc Netw.* 2007;5(1):3-7.

© 2020 Shahrokni A et al. *JAMA Network Open.*

eTable 1. The Electronic Rapid Fitness Assessment Instruments

Domain, Instrument	**Description**	**Scoring**	**Abnormal Score**
Functional Status			
Basic Activities of Daily Living[3-5]	Level of independence in 7 activities: bathing, dressing, grooming, feeding, bladder control, walking inside the home, and walking outside the home	Points scored for each rated activity.<br>Limited a lot: 0 points<br>Limited a little: 1 point<br>Not limited at all: 2 points<br>Higher scores relate to better independence.	<14
Instrumental Activities of Daily Living[6,7]	Level of independence in 8 activities: telephone use, doing laundry, shopping, preparing meals, doing housework, handling own medication, handling money and finances, and transportation	Points scored for each rated activity.<br>Unable to perform the activity: 0 points<br>Needed some help: 1 point<br>Did not need any help: 2 points<br>Higher scores relate to better independence.	<16
Patient-Rated Karnofsky Performance Score[8]	Rating of performance status	Between 100-30 in 10-point increments.<br>Higher scores relate to better performance.	≤80
Timed Up and Go test[9-11]	Patient is asked to stand from chair, walk 10 feet, turn, and return to chair while timed	<10 seconds, 10-19 seconds, ≥20 seconds	≥10 seconds; patients who were unable to get up from chair without use of their arms
Falls	Total number of falls within the last year	None, 1 time, >1 time	≥1 fall within the last year
Nutrition status, weight change	Weight change within past 6 months	No weight change or weight gain, <5 lb weight loss, 5-10 lb weight loss, 10-20 lb weight loss, >20 lb weight loss	≥10 lb weight loss
Polypharmacy, polypharmacy[12]	Total number of prescribed medications	0, 1-4, 5-10, >10 medications	≥5 medications
Cognition, Mini-Cog[1]	Patient asked to recall 3 words and perform clock draw test	2 points scored for correct clock draw; 1 point scored for each word recalled.	≤2
Social Support, Four-item Medical Outcomes Study-Social Support Survey[13]	Four 5-point Likert scale questionnaire on availability of emotional/informational, tangible, affectionate, and positive social interaction	Score can be 1-5 points per item; total score ranges from 4 to 20.<br>Higher scores relate to more social support.	≤16
Social Activity Interference, Medical Outcome Study-	Three 5-point Likert scale	Score can be 1-5 points per item; total score	≥8

© 2020 Shahrokni A et al. *JAMA Network Open.*

Social Activity Survey[14]	questionnaire on the interference of patient's health condition with the social activity	ranges from 3 to 15.<br>Higher scores relate to higher social activity limitation.	
Emotional Status			
Geriatric Depression Scale 4-item[15]	Four-item yes/no questionnaire of patient's psychological status	1 point per question	Score ≥1
Distress Thermometer[16-20]	Patient asked to rate distress level in last 2 weeks	Between 10 (extreme distress) and 0 (no distress)	Score ≥4

© 2020 Shahrokni A et al. *JAMA Network Open.*

eTable 2. Proportion of Patients Referred for Geriatric Comanagement Based on Procedure Type

**Procedure type**	**Surgical Service Management (N=872)**	**Geriatric Comanagement (N=1020)**
Colorectal	137 (23)	448 (77)
Gastric and mixed tumor	92 (53)	82 (47)
Gynecology	45 (15)	264 (85)
Head and neck	147 (32)	314 (68)
Urology	78 (30)	180 (70)
Plastic	47 (28)	118 (72)
Hepato-biliary-pancreatic	55 (23)	183 (77)
Thoracic	309 (67)	150 (33)
Other procedures	109 (41)	159 (59)

Data are no. (%). Percentages are proportions of the procedure.

© 2020 Shahrokni A et al. *JAMA Network Open.*

eTable 3. Association Between Geriatric Comanagement and Mortality on Sensitivity Analyses[a]

**Outcome**	**Odds Ratio (95% CI)**	***P***
Death within 30 days (N=1892)	0.36 (0.18-0.73)	0.005
Death within 60 days (N=1892)	0.36 (0.22-0.60)	<0.0001

[a]All analyses are adjusted for age at surgery, operative time, preoperative albumin level, and Memorial Sloan Kettering Frailty Index score. The outcome of death within 60 days was additionally adjusted for sex, American Society of Anesthesiologists score, and estimated blood loss.

© 2020 Shahrokni A et al. *JAMA Network Open.*

eTable 4. Association Between Geriatric Comanagement and 90-day Mortality on Sensitivity Analyses, With Adjustment for Additional Covariates

**Model**	**Odds Ratio (95% CI)**	***P***
Additionally, adjusted for time to surgery (N=1892)	0.47 (0.30-0.73)	0.001
Additionally, adjusted for preoperative hemoglobin level (N=1606)	0.49 (0.30-0.80)	0.004
Additionally, adjusted for preoperative sodium level (N=1604)	0.50 (0.31-0.82)	0.006
Additionally, adjusted for preoperative calcium level (N=1602)	0.49 (0.30-0.80)	0.004
Additionally, adjusted for preoperative hemoglobin, preoperative sodium, and preoperative calcium levels (N=1600)	0.49 (0.30-0.80)	0.005
Additionally, adjusted for heart disease and arthritis or degenerative joint disease (N=1892)	0.47 (0.30-0.74)	0.001
Additionally, adjusted for procedure type (N=1892)	0.57 (0.36-0.90)	0.015
Additionally, adjusted for procedure type and time to surgery (N=1892)	0.58 (0.36-0.91)	0.018
Additionally, adjusted for procedure type, time to surgery, heart disease, and arthritis or degenerative joint disease (N=1892)	0.58 (0.37-0.92)	0.021

All analyses are adjusted for age at surgery, sex, American Society of Anesthesiologists score, operative time, preoperative albumin level, estimated blood loss, and Memorial Sloan Kettering Frailty Index score.

© 2020 Shahrokni A et al. *JAMA Network Open.*

eTable 5. Adverse Surgical Events Within 30 days of Surgical Treatment

**Event**	**Surgical Service Management (N=872)**	**Geriatric Comanagement (N=1020)**
Major complication	37 (4)	72 (7)
Readmission	115 (13)	104 (10)
Emergency room visit	151 (17)	150 (15)

Data are no. (%).

© 2020 Shahrokni A et al. *JAMA Network Open.*
